# Surgical, Dermatological and Morphopathological Considerations in the Diagnosis and Treatment of Cutaneous Lymphoproliferative Tumors, Primary CD4+

**DOI:** 10.3390/medicina58111618

**Published:** 2022-11-10

**Authors:** Valeriu Ardeleanu, Lavinia-Alexandra Moroianu, Anca Sava, Tiberiu Tebeica, Radu Cristian Jecan, Marius Moroianu, Alin Laurentiu Tatu

**Affiliations:** 1 Doctoral School, Faculty of Medecine, “Ovidius” University, 1 University Alley Street, Corp B, 900470 Constanta, Romania; 2 General Hospital “Căi Ferate”, 4-6 Alexandru Morutzi Street, 800223 Galați, Romania; 3 Arestetic Clinic, 78 Brailei Street, BR4A, 800108 Galați, Romania; 4 Faculty of Kinesiotherapy, University “Dunărea de Jos”, 47 Domnească Street, 800008 Galați, Romania; 5 “Elisabeta Doamna” Psychiatry Hospital, 290 Traian Street, 800179 Galați, Romania; 6 Clinical Medical Department, Faculty of Medicine and Pharmacy, “Dunărea de Jos” University, 47 Domnească Street, 800008 Galați, Romania; 7 Department of Morpho-Functional Sciences I, Faculty of Medicine, University of Medicine and Pharmacy “Gr. T. Popa”, 16 Universitatii Street, 700115 Iasi, Romania; 8 Department of Pathology, “Prof. Dr. Nicolae Oblu” Emergency Clinical Hospital, 2 Ateneului Street, 700309 Iasi, Romania; 9 Leventer Medical Center, 8 Monetariei Street, 011216 Bucharest, Romania; 10 Department of Plastic Surgery and Reconstructive Microsurgery, “Carol Davila” University of Medecine and Pharmacy, 8 Eroii Sanitari Street, 050474 Bucharest, Romania; 11 Clinical Department of Plastic Surgery and Reconstructive Microsurgery, “Prof. Dr. Agrippa Ionescu” Emergency Clinical Hospital, 7 Architect Ion Mincu Street, 011356 Bucharest, Romania; 12 Department of Dental Medicine, Faculty of Medicine and Pharmacy, “Dunărea de Jos” University, 47 Domnească Street, 800008 Galați, Romania; 13 Medical Assistance Service of the Municipality of Galați, 97 Traian Street, 006757 Galați, Romania; 14 Dermatology Department, “Sfanta Cuvioasa Parascheva” Clinical Hospital of Infectious Diseases, 393 Traian Street, 800179 Galați, Romania; 15 Multidisciplinary Integrated Center of Dermatological Interface Research MIC-DIR (Centrul Integrat Multidisciplinar de Cercetare de Interfata Dermatologica—CIM-CID), “Dunărea de Jos” University, 47 Domnească Street, 800008 Galați, Romania

**Keywords:** cutaneous lymphoma, immunohistochemistry, dermoscopy, cutaneous tumor

## Abstract

Primary cutaneous lymphomas are a heterogeneous group of T-cell (CTCL) and B-cell lymphomas (CBCL) developing in the skin and without signs of extracutaneous disease at the time of diagnosis. The term “primary small/medium CD4+ T-cell lymphoma” was changed to “primary small/medium cutaneous CD4+ lymphoproliferative disorder” due to its indolent clinical behavior and uncertain malignant potential. This paper presents a rare case of primary cutaneous lymphoma with small to medium CD4+ T-cells. A 37-year-old patient presented with a tumor in the frontal region that had occurred approximately 8–9 months earlier. The tumor had a diameter of about 8–9 mm, well demarcated macroscopically, it was round in shape, about 6–7 mm high, pink in color, firm in consistency and painless during palpation. Surgical excision of the tumor was performed with a margin of safety of 8 mm and deep to the level of the frontal muscle fascia. The histopathological examination supported the diagnosis of cutaneous lymphoproliferation with a nodular disposition in the reticular dermis and extension around the follicular epithelia and sweat glands, composed mainly of dispersed medium-large lymphocytes. Additional immunohistochemical examination was requested. Immunohistochemical examination confirmed the diagnosis of “primary cutaneous CD4+ small/medium T-cell lymphoproliferative disorder.” Patient monitoring was carried out through clinical dermatological controls at 3, 6, and 12 months. After one year, a cranio-cerebral MRI was performed. For the following 5 years, an annual dermatological examination accompanied by cranio-cerebral MRI, blood count, and pulmonary X-ray were recommended. Similarly to all solitary skin lesions, the prognosis is excellent in this case, the only treatment being surgical excision.

## 1. Introduction

Primary cutaneous lymphomas are a heterogeneous group of T-cell (CTCL) and B-cell lymphomas (CBCL) that occur in the skin, with no extracutaneous disease signs at the time of diagnosis. CTCLs represent about 75–80% of all primary skin lymphomas, and CBCL account for 20–25%. In September 2018, an updated version of the WHO-EORTC (World Health Organization—European Organization for Research and Treatment of Cancer) was published, in which primary cutaneous CD8+ T-cell lymphoma and Epstein-Barr virus positive (EBV+) mucocutaneous ulcer were included as new temporary entities and a new section on cutaneous forms of chronic active EBV disease was added. The term “primary cutaneous small/medium CD4+ T-cell lymphoma” was changed to “primary cutaneous small/medium CD4+ lymphoproliferative disorder” due to its indolent clinical behavior and uncertain malignant potential. WHO-EORTC stated a 6% frequency of primary cutaneous CD4+ small to medium T-cell lymphoproliferative disorder from the CTCL group. Due to the high rate of healing (100% for a 5 year period) and the good prognosis, they were included among lymphoproliferative disorders and not lymphomas. Changes were also made to the lymphomatoid papulosis sections; thus, increasing the spectrum of the histological and genetic types of primary cutaneous lymphomas in the marginal zone, and recognizing two different subtypes [1].

The differential diagnosis is made with other primary lymphomas featuring T- or B-cells, or those that have deterministic effects on the skin, such as pseudo-lymphomas, Lupus Tumidus, or Jessner–Kanoff lymphocytic infiltrate.

From a treatment perspective, incisional or excisional biopsy can be considered the first stage. In some cases, low-dose radiotherapy can be considered a therapeutic option, but this has the disadvantage of possible regional adverse reactions. In cases of folliculitis or other overlapping dermatoses, the dermatological procedure is addressed. Phototherapy or light treatment (UVA and UVB) may also be an option.

The study of primary cutaneous lymphomas is far from over, particularly in the field of immunohistochemistry, where research is being carried out; thus, other techniques for the study of malignant tumors may be extrapolated to this type of tumor [2].

## 2. Case Report

**Figure 1:**
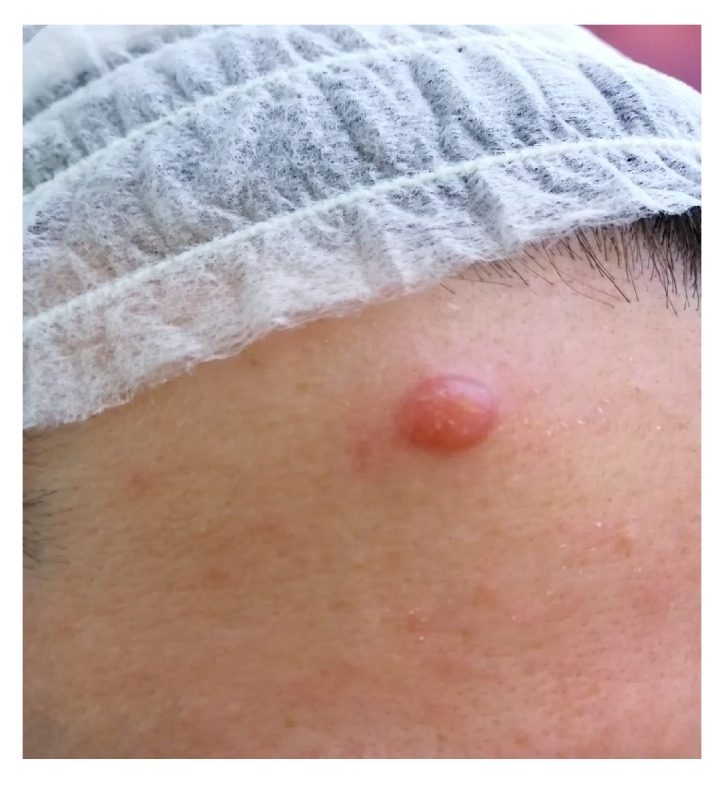
Primary lesion before surgical treatment.

We present the case of a 37-year-old asymptomatic female patient with a tumor in the frontal region, which appeared about 8–9 months ago. The tumor had a diameter of about 8–9 mm, macroscopically well-delimited, it was round in shape, of about 6–7-mm in height, pink, firm in consistency and not painful during palpation. In the perilesional skin, but also discreetly on the skin surface of the tumor, white-yellowish spicules were observed (Figure 1), confirmed by dermoscopy as Demodex tails belonging to *Demodex folliculorum*. Following the dermatological consultation, but also for aesthetic reasons, it was decided to surgically remove the tumor.

Surgical excision of the tumor was performed with a safety limit of 8 mm and deep to the level of the frontal muscle fascia. The wound was sutured in two planes, a 3.0 deep absorbable suture and a 4.0 non-absorbable transcutaneous suture, removed after 14 days. The postoperative evolution was favorable, without any complications.

**Figure 2:**
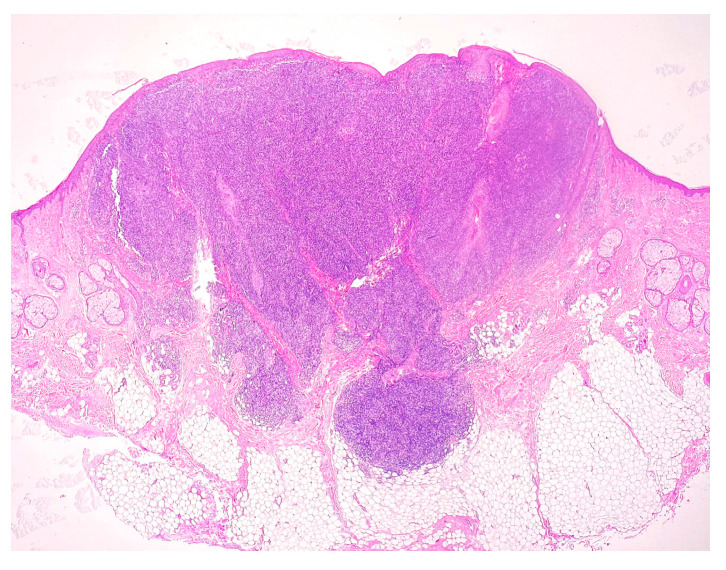
HE staining. Magnification 20×.

**Figure 3:**
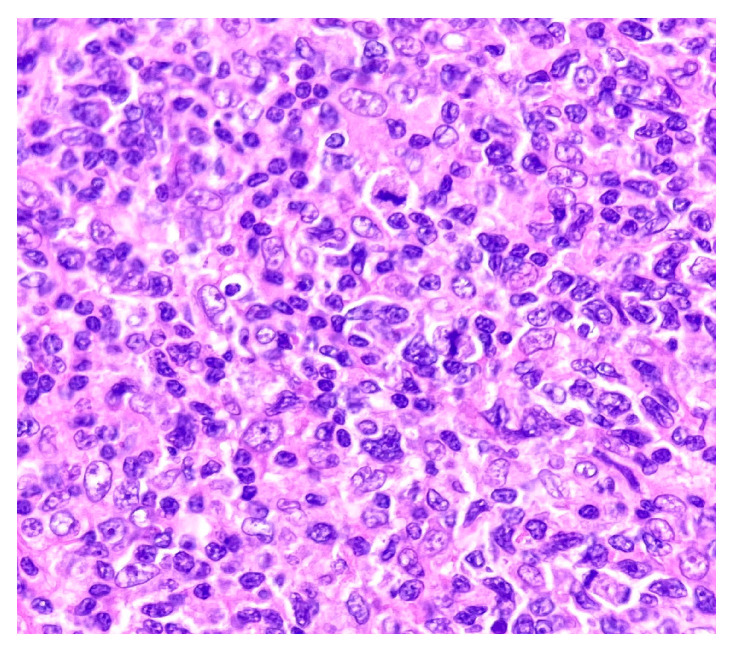
HE staining. Magnification ×400.

The histopathological examination supported the diagnosis of cutaneous lymphoproliferation with nodular disposition in the reticular dermis and extension around the follicular epithelia and sweat glands (Figure 2), composed mainly of dispersed medium-large lymphocytes, with vesicular nuclei, some surrounded by peripherally located small lymphocytes arranged in a string of pearls (Figure 3). Small intralesional groups of epithelioid histiocytes and an infiltrate with small reactive lymphocytes and peripheral plasma cells were present; the supralesional epidermis was not affected. The excision was complete in all examined planes. Additional immunohistochemical examination was requested.

The immunohistochemical examination confirmed the diagnosis of “primary cutaneous CD4+ small/medium T-cell lymphoproliferative disorder.” The immunohistochemical markers are presented in Table 1.

**Figure 4:**
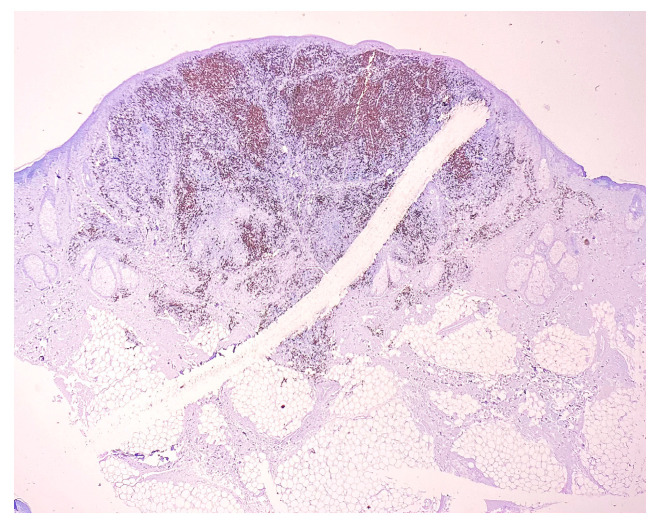
CD20 staining. Magnification 20×.

The immunohistochemical markers examined were:

CD20: positive in 30–35% of infiltrate lymphocytes (B lymphocytes), fairly evenly dispersed (Figure 4). CD20 is a nonglycosylated phosphoprotein member of the MS4A family, which forms a tetraspan membrane-bound protein and is a B-cell marker. CD20 expression is present at the pre-B-cell stage and disappears upon its differentiation into a plasma cell [3]. CD20 acts as a calcium channel localized to lipid rafts, but the specific function of CD20 remains unknown. CD20 has dynamic cellular activity and remains a specific therapeutic target for B-cell malignancies [4].

**Figure 5:**
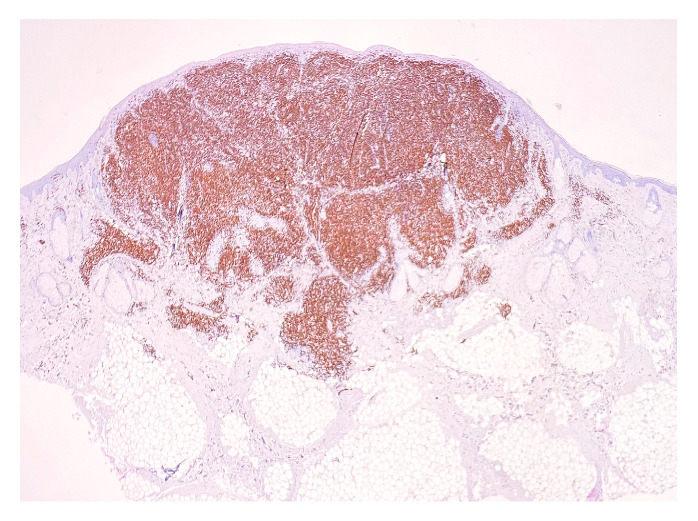
CD3 staining. Magnification 20×.

CD3: positive in 65–70% of infiltrate lymphocytes (T lymphocytes). This highlights the extension of the infiltrate around the appendages, without aspects of epidermotropism (Figure 5). The antigen remains present in all T-cell leukemias and lymphomas, being used to distinguish them from similar B-cell neoplasms [5].

**Figure 6:**
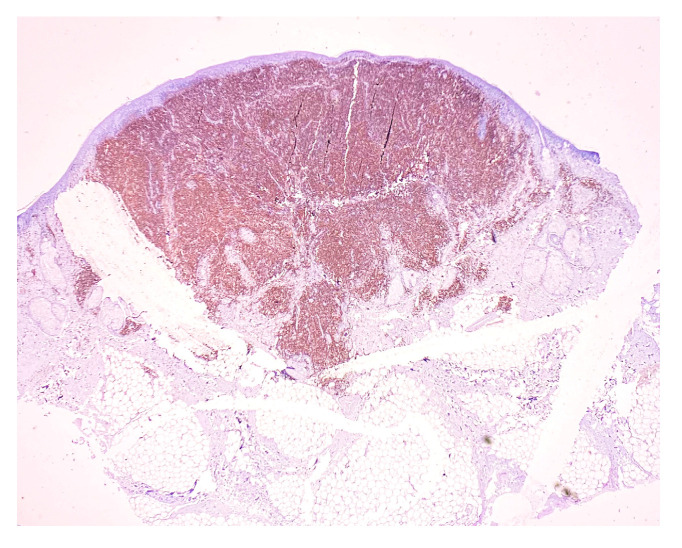
CD4 staining. Magnification 20×.

CD4: positive in most infiltrate lymphocytes (T helper lymphocytes) (Figure 6). CD4+ T helper cells represent an essential part of the human immune system. They are often referred to as T helper or T4 cells. CD4 immunohistochemistry is useful for biopsy samples to identify peripheral T-cell lymphoma and related malignant diseases. The antigen has been associated with autoimmune diseases, such as diabetes mellitus type I and vitiligo [6]. CD4+ T helper cells have a significant function in autoinflammatory diseases and chronic infections [7].

**Figure 7:**
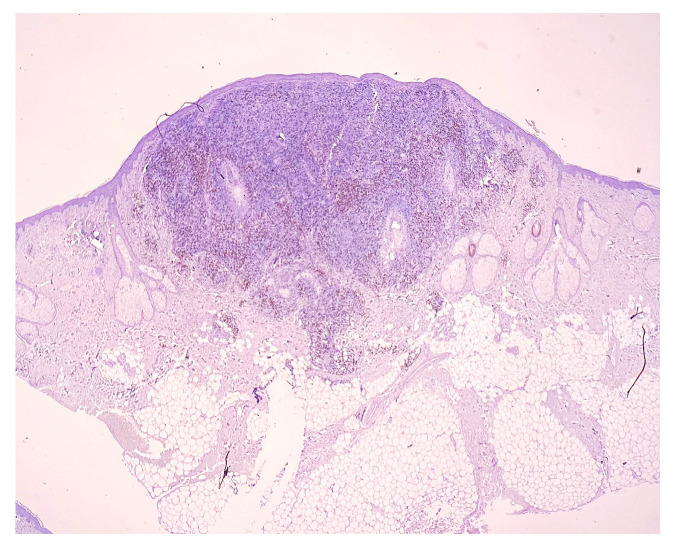
CD8 staining. Magnification 20×.

CD8: positive in dispersed T lymphocytes (T cytotoxic lymphocytes) (Figure 7). CD8+ T-cells (often called cytotoxic T lymphocytes) are important for immune defense against intracellular pathogens as well as for tumor surveillance [8].

CD30: positive in dispersed activated lymphocytes, it is associated with anaplastic large cell lymphoma, and is expressed in embryonal carcinoma but not in seminoma; thus, being a useful marker in distinguishing between these germ cell tumors. CD30 is also expressed on Reed–Sternberg cells, which are typical for Hodgkin’s lymphoma [9].

**Figure 8:**
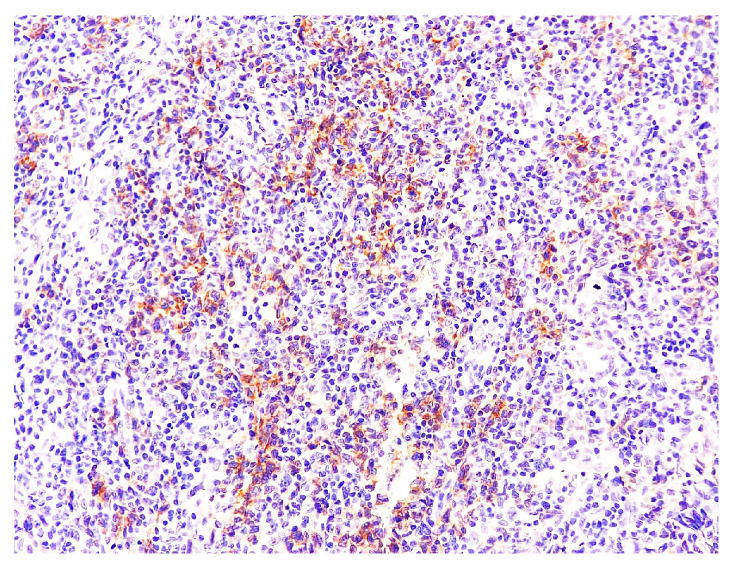
PD-1 staining. Magnification 200×.

**Figure 9:**
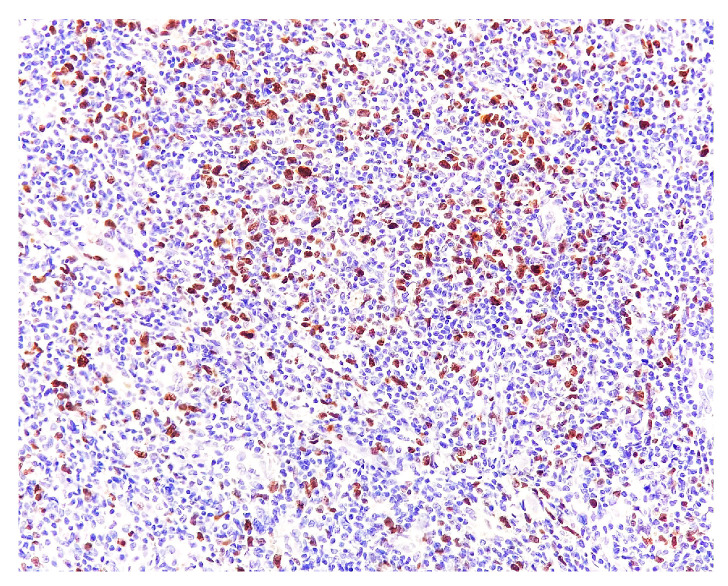
Ki67 staining. Magnification 200×.

PD-1: positive in many infiltrate cells, including slightly pleomorphic lymphoid cells with a hypertrophic nucleus (T follicular helper lymphocytes) (Figure 8). PD-1 guards against autoimmunity via two mechanisms: it promotes apoptosis of antigen-specific T-cells in lymph nodes and reduces apoptosis in regulatory T-cells [10]. Ki67: positive in 20% of the infiltrate cells (Figure 9).

Patient monitoring was carried out through clinical dermatological controls at 3, 6, and 12 months. At 1 year, a cranio-cerebral MRI was performed. For the following 5 years, an annual dermatological examination accompanied by a cranio-cerebral MRI, blood count, and pulmonary X-ray were recommended.

Similarly to all solitary skin lesions, the prognosis is excellent in this case, the only treatment being surgical excision.

## 3. Discussion

According to the European Society of Medical Oncology Clinical Practice Guideline for primary cutaneous lymphoma (published in June 2018), in the Western population, CTCL represents 75–80% of all primary cutaneous lymphomas (mycosis fungoides (MF) being the most common type), and CBCL represents 20–25%. However, the rate of its occurrence is different in other parts of the world. For example, in Southeast Asia, CTCLs, except for MF, are much more common than in Western countries, and CBCLs are much less common. Two of the most common types of CTCL are MF and Sézary syndrome (SS). The other forms of CTCL include CD30+ primary cutaneous lymphoproliferative diseases; subcutaneous T-cell lymphoma with panniculitis appearance; NK/T-cell extranodal lymphoma, nasal type (very rare in Western countries, but very common in Asia, as well as Central and South America); and primary cutaneous lymphoma with peripheral T-cells—unspecified type. Rare types of cutaneous T-cell lymphoma are primary cutaneous CD4+ small to medium T-cell lymphoma, primary cutaneous gamma/delta T-cell lymphoma, and primary aggressive cutaneous CD8+ cytotoxic epidermotropic T-cell lymphoma.

Primary cutaneous CD4+ small to medium T-cell lymphoma may begin asymptomatically, as solitary or multiple micro-papules, micro-nodes, or plaques, and are often purplish-pink with no distinct individual clinical features and are difficult to differentiate from other similar incipient nodular lesions [11].

A challenge associated with reliably diagnosing cutaneous lymphoma is that its signs and symptoms are not the same for all patients, and some of the symptoms, especially when they are milder, are often mistaken for conditions such as eczema or psoriasis, fungal skin reactions (such as ringworm), various skin reactions caused by medicines, certain substances, or allergies. Spots, plaques, papules, or tumors are clinical names for various skin symptoms (also known as lesions) that can be clues to diagnosis.

Of the very few cases reported in the literature, those located on the face have been rarely described and those on the trunk and limbs are more often found in children than in adults [12]. Some studies present a controversial association with the existence of a co-infection with B. Burgdorferi, unconfirmed in our case, or consistent with other autoimmune diseases or post-vaccine reactions [13,14]. Most often, lymphoproliferative cutaneous tumor CD4+ remains asymptomatic; it evolves, but it is difficult to predict its development. The apparently hyperkeratosis spicules rarely described in the literature, which we also observed clinically and by using dermoscopy, are in fact indicators of the tails of *Demodex folliculorum* (DF). Demodex tails can multiply in areas of possible perilesional immunosuppression and less directly on the cutaneous area overlying the nodule. This is due to the reduction in length of the follicular duct by underlying hyper pressure, which prevents DF development; a phenomenon also described by Tatu et al., in other works, as an early marker of the development of underlying lesional hyper pressure, on the one hand, and as a zonal, perilesional immunosuppressive factor, on the other hand. Although not present in the patient, coexistence in the early stages of a violaceous erythematous nodule with early papular nodular lesions of rosacea may complicate the clinical diagnosis, which involves the use of other dermatological imaging techniques, such as Canfield Visia skin analysis, in vivo confocal microscopy, cutaneous optical coherence tomography, and dermoscopy [15].

In terms of dermoscopy, the literature presents few descriptions of dermoscopic imaging in lymphoproliferative cutaneous tumor CD4+. In this instance, the dermoscopy revealed a salmon-colored background and serpentine blood vessels; thus, entering the differential diagnosis of basal cell carcinoma, inflamed intradermal nevus, achromic melanoma, reaction to insect bite, and sarcoidosis, etc. [16,17].

From a clinical perspective, other diagnoses, such as facial granuloma, other primary T-cell or B-cell lymphomas, or skin deterministic effects, such as pseudolymphomas, Lupus Tumidus, or Jessner–Kanoff lymphocytic infiltrate may be considered [18,19,20].

From an immunohistochemical perspective, the primary cutaneous CD4+ small to medium T-cell lymphoma is a rare lymphoma (2% of all cutaneous T-cell lymphomas). It is characterized by the predominance of small to medium sized CD4+ pleomorphic T-cells without evidence of the typical plaques of MF.

Patients present with a solitary plaque or nodule on the face, neck, or upper trunk, the involvement of the lower extremities being rare. These plaques are asymptomatic, the only clinical feature being a small solitary skin lesion.

The histological features of this type of lesion are represented by a dense, diffuse, or nodular infiltrate of the skin with a tendency to subcutaneous involvement. The infiltrate is formed by small to medium sized pleomorphic T-cells, sometimes with a small proportion of large pleomorphic cells. In addition, the infiltrate may contain reactive lymphocytes, histiocytes, eosinophils, or plasma cells [21]. In this instance, it was positive for CD3 and CD4.

In terms of treatment, a positive prognosis and localized proliferation makes surgical treatment a good option, with complete excision followed by sutures and/or reconstructions possible, depending on the location and the particularities of the case. Although an exploratory incisional biopsy can be performed, we recommend complete excision per primam, followed by histopathological and immunohistochemical examinations. We do not consider useful the expectant attitude recommended by some authors, because the nodule often progresses in time and the excision area becomes wider (20). In specific cases, low-dose radiotherapy may be an alternative, but regional adverse reactions may limit this procedure. Local and intralesional treatments have not proven to be effective. In cases of folliculitis or other overlapping dermatosis, the dermatological procedure is addressed. In the particular case of this patient, topical treatment with Ivermectin-containing cream, following the healing of the post-excision scar, led to the disappearance of the DF spicules. This attitude is important, both from a curative and preventive perspective; especially since it has been recently described that *Demodex folliculorum* may be a factor in determining other viral infections, including SARS-CoV-2. This is not only from the perspective of chitin–lipid interactions but also of immunosuppression associated with lymphoma patients, as well as the activation of inflammatory, immunological and, possibly, proliferative phenomena by the endosymbionts attached to *Demodex folliculorum* [22,23]. In addition, and for further evolution, we consider the emotional factors that exist in patients with chronic diseases, and especially in those with aesthetic affectation [24]. The use of steroids, interferons, HDAC inhibitors, or chemotherapy can be a therapeutic option, but we do not consider it necessary in this type of cutaneous lymphoma at this stage.

## 4. Conclusions

The particularity of this case report is represented by a rare case of cutaneous T-cell lymphoma, more accurately described as primary cutaneous CD4+ small to medium T-cell lymphoma, that has an overtly benign clinical course. The condition has an excellent prognosis and responds well to skin-directed therapies. Practitioners should be aware of this condition to avoid aggressive systemic treatments.

The histopathological features of this lesion are represented by a dense, diffuse, or nodular infiltrate of the skin. The infiltrate is formed by small to medium sized pleomorphic T-cells with a small proportion of large pleomorphic cells.

Solitary skin lesions have an excellent prognosis (surgical excision).

The particularity of this case is represented by this rare type of tumor.

## Figures and Tables

**Table 1:** The immunohistochemical markers.

Antibody	Clone	Producer	Pretreatment	Dilution
CD3	MRQ-39	Cell Marque	HIER EDTA 19 min	1:100
CD4	EP204	Cell Marque	HIER EDTA 19 min	1:200
CD8	C8/144B	Cell Marque	HIER EDTA 19 min	1:200
CD20	L26	Cell Marque	HIER EDTA 19 min	1:100
CD30	Ber-H2	Bio SB	HIER EDTA 19 min	1:100
Ki67	MIB-1	DAKO	HIER EDTA 19 min	1:200
PD1	NAT105	Cell Marque	HIER EDTA 19 min	1:100

HIER = heat induced epitope retrieval.

## Data Availability

Not applicable.
